# *In situ* stress observation in oxide films and how tensile stress influences oxygen ion conduction

**DOI:** 10.1038/ncomms10692

**Published:** 2016-02-25

**Authors:** Aline Fluri, Daniele Pergolesi, Vladimir Roddatis, Alexander Wokaun, Thomas Lippert

**Affiliations:** 1 Department for Energy and Environment, Paul Scherrer Institut, 5232 Villigen-PSI, Switzerland; 2 Institut für Materialphysik, Universität Göttingen, Friedrich-Hund-Platz 1, Göttingen 37077, Germany; 3 Department of Chemistry and Applied Biosciences, Laboratory of Inorganic Chemistry, Vladimir-Prelog-Weg 1-5/10, ETH Zürich, Zürich CH-8093, Switzerland

## Abstract

Many properties of materials can be changed by varying the interatomic distances in the crystal lattice by applying stress. Ideal model systems for investigations are heteroepitaxial thin films where lattice distortions can be induced by the crystallographic mismatch with the substrate. Here we describe an *in situ* simultaneous diagnostic of growth mode and stress during pulsed laser deposition of oxide thin films. The stress state and evolution up to the relaxation onset are monitored during the growth of oxygen ion conducting Ce_0.85_Sm_0.15_O_2-δ_ thin films *via* optical wafer curvature measurements. Increasing tensile stress lowers the activation energy for charge transport and a thorough characterization of stress and morphology allows quantifying this effect using samples with the conductive properties of single crystals. The combined *in situ* application of optical deflectometry and electron diffraction provides an invaluable tool for strain engineering in Materials Science to fabricate novel devices with intriguing functionalities.

## Abstract (web summary)

Strain engineering is used to tune physiochemical material properties, but detailed insights of how the crystal growth affects the stress are yet lacking. Here, the authors analyse in situ simultaneously the induced stress and growth mode during the epitaxial growth of an oxygen ion conductor.

## Graphical abstract

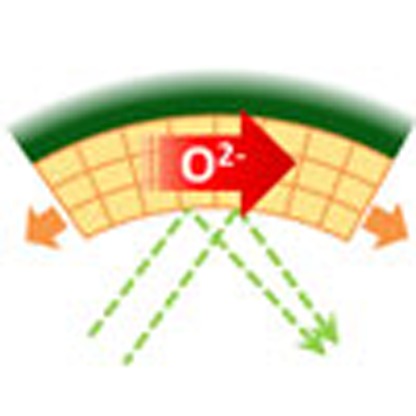

Stress-induced lattice distortions, that is, a tensile or compressive strain of the crystal structure, can significantly influence the physicochemical characteristics of materials by enhancing/inhibiting specific properties or by enabling new functionalities not allowed in the unperturbed structure. Strain engineering offers indeed a new route to tune the characteristics of a material for example by modifying the electronic bandgap^1^, multiferroic^2^ or catalytic^3^ properties, thermal conductivity^4^ and charge transport^5^,^6^,^7^,^8^.

To investigate the effects of strain, epitaxial thin films are typically used where the strain is induced by a lattice mismatch at the interface with the substrate. For highly ordered epitaxial films, established models exist, which assume elastic distortions and coherent growth, that is, a 1:1 matching between all lattice planes^9^,^10^,^11^. The upper limit of the strain is in this case the lattice mismatch between film and substrate. In general, however, we often deal with semi-coherent heterointerfaces where it is energetically favourable to reduce the elastic energy by introducing dislocations. Already a mismatch exceeding ∼1% is typically not expected to be fully accommodated^9^,^10^,^11^. The growth mode (layer-by-layer or island-like) also influences the effective strain through the surface morphology and relaxation behaviour^12^,^13^,^14^.

Monitoring the stress and the growth mode *in situ* (during the growth) allows investigating fundamental mechanisms of stress generation and evolution from the initial nucleation to the onset of relaxation. Literature concerning the *in situ* diagnostic of stress/strain generation and evolution during the epitaxial growth of oxide films is scarce. Here, we address this topic measuring simultaneously *in situ* the stress with a multi-beam optical stress sensor (MOSS) and the growth mode by reflection high-energy electron diffraction (RHEED) during pulsed laser deposition (PLD).

When a stressed film grows, the substrate bends minimizing the elastic energy. Stoney's equation^10^ correlates the substrate curvature 1/*ρ* with the film stress *σ* as


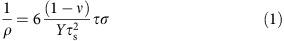


where *ρ* being the ray of curvature, *τ* and *τ*_s_ the film and substrate thicknesses, *ν* and *Y* the Poisson ratio and the Young's modulus of the substrate and *σ* the average film stress. As sketched in Fig. 1, the MOSS uses the deflection of laser beams to measure the change in curvature, and thus to monitor *in situ* and in-plane the stress of the growing film^10^. In contrast, conventional X-ray diffraction analysis yields the strain, *ex situ* and out-of-plane.

The in- and out-of-plane strain of a film, *ɛ*_*xx*_ and *ɛ*_*zz*_, are related through the Poisson ratio^9^ *ν* as


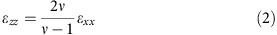


For most materials 0<*v*<0.5, implying that an in-plane tensile (compressive) strain of the unit cell corresponds to an out-of-plane compressive (tensile) strain.

Different *in situ* strain diagnostics have been applied for thin films. The strain evolution of Ba_0.5_Sr_0.5_TiO_3_ thin films grown on MgO was investigated using real-time X-ray diffraction^15^. This is undoubtedly a very powerful approach but it requires a synchrotron light source^16^ precluding its application as a routine measurement technique. RHEED was also employed to monitor the in-plane strain evolution, for example, during the growth of BaTiO_3_ on SrTiO_3_ (refs 9, 14). This specific application of RHEED is very rare, even considering the widespread use of high-pressure RHEED. In fact, to minimize electron scattering, it can only be attempted at low pressure, a growth condition unsuitable for many oxides. A wafer curvature measurement similar to MOSS was used during PLD of BaTiO_3_ and SrTiO_3_ ultra-thin films on a Pt cantilever^17^. The main advantages of MOSS over similar deflection techniques are that one can use any substrate, no position sensitive detector is needed and that the noise is reduced through multiple beams^10^.

Several examples of MOSS (or similar techniques) applications for studying the stress evolution during the growth of semiconductor or metal thin films exist^18^,^19^,^20^,^21^,^22^,^23^,^24^, whereas reports on MOSS diagnostic with oxide materials are very rare^25^. To our knowledge, this technique has never been applied simultaneously with RHEED during PLD, the most widespread method for the growth of oxide films. In particular, by coupling the MOSS with RHEED, the stress state of the growing film can be correlated with the growth mode from the initial nucleation to stress relaxation, thus providing a unique diagnostic tool.

15 at.% Sm-doped CeO_2_ (SDC) is the material selected for this fundamental study. SDC is an oxygen-ion conductor used as electrolyte in solid oxide fuel cells (SOFCs), environmentally sustainable electrochemical energy converters^26^. The oxygen ion conductivity *σ*_ion_ is described with the Arrhenius equation


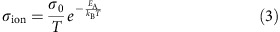


*T* being the temperature, *σ*_0_ the pre-exponential factor, *E*_A_ the activation energy and *k*_B_ the Boltzmann constant. Currently, the most accepted hypothesis is that tensile strain lowers the activation energy for the oxygen-ion hopping by increasing the migration volume^7^,^8^,^27^. Consequently, larger ionic conductivities could be achieved at lower temperatures, with important consequences for technological applications.

Here, as a case study for the first MOSS plus RHEED application during PLD of oxide materials, the fundamental question of how strongly homogeneous strain can affect the ionic conductivity in a single crystal is addressed. This fundamental question is of interest as controversial results have been reported on the magnitude of the effect of strain on the ionic conductivity^8^,^27^,^28^. The increase in conductivity ascribed to strain effects spreads over several orders of magnitude showing the need to evaluate the effect using samples where any potential side effect is minimized and where the strain/stress state is unambiguously identified.

To achieve this goal, thin films of high crystalline quality—as close to a single crystal as experimentally possible—but with different strain values are employed as model systems. The MOSS is particularly suited for this investigation as the stress state can be probed in-plane, precisely in the direction of the ion migration during conductivity measurements, and *in situ* in a temperature range similar to the operating temperature of SDC for technological applications. This study confirms that tensile strain indeed leads to a decrease in activation energy showing that the effect is relatively small, roughly a factor of 2 at around 350 °C. But more importantly, we show here the potentials of MOSS diagnostic as an invaluable tool for strain engineering in Materials Science.

## Results

### Strain generation and evolution on different substrates

**Figure 2:**
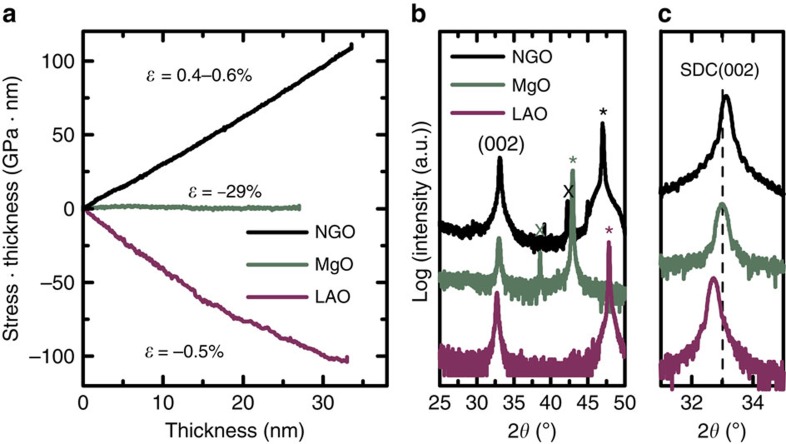
MOSS and XRD analysis of SDC films grown on NGO and MgO and LAO. (**a**) Stress-thickness product, evaluated using the Stoney equation and the elastic properties of the substrates^57^,^58^,^59^, as a function of thickness. A positive (negative) stress value indicates an in-plane tensile (compressive) stress for the film. *ɛ* is the lattice mismatch (Table 1). (**b**) *ω*/2*θ* scans. *indicates the substrate peak and X is an instrumental artefact (*K*_β_ reflection of substrate). (**c**) Magnification of the region around the (002) diffraction peak of SDC. The dashed line indicates the angular position for the fully relaxed SDC structure. The peak shifts are in accordance with the sign of the stress-thickness product.

To study the strain generation and evolution in SDC films, MgO, NdGaO_3_ (NGO) and LaAlO_3_ (LAO) single-crystal substrates were used; Table 1 reports their crystallographic properties, the expected epitaxial relations and the in-plane lattice misfit with 15 at.% SDC (cubic fluorite structure with a lattice parameter of 5.43 Å). NGO and LAO provide a lattice mismatch in a suitable range to expect epitaxial films of SDC in tensile and compressive in-plane strain, respectively. On the contrary, the large lattice mismatch between SDC and MgO may prevent any crystallographic matching leading to a fully relaxed growth from the start. Figure 2 compares the MOSS *in situ* stress characterizations with the X-ray diffraction *ex situ* strain measurements of (001)-oriented SDC films on the different substrates. The MOSS measurements are reported as stress-thickness product (directly proportional to the curvature, from the Stoney equation (1)) in function of thickness, a plot commonly used in the literature for wafer curvature measurements^19^,^20^,^21^,^22^,^23^,^24^,^25^.

As expected, during the growth on MgO, the stress-thickness product, that is, the substrate curvature, remains constant indicating that the film grows without exerting any force on the substrate, that is, stress free. Correspondingly, the X-ray diffraction analysis shows a fully relaxed crystalline structure. The large lattice misfit is accommodated by a high density of interfacial misfit dislocations that fully release the stress as revealed by transmission electron microscopy, discussed below. For the SDC film grown on NGO, the positive curvature indicates that the film exerts a compressive force on the substrate surface and bends it: the film is under in-plane tensile stress, which originates at the very beginning of the growth. The constant slope of the stress-thickness product (Fig. 2a) shows that the stress is constant to a film thickness of 33 nm. An average stress of ∼3.3 GPa can be calculated. The tensile in-plane stress is in agreement with the compressive out-of-plane strain of −0.52% measured *ex situ* by XRD. The XRD analysis of the 33-nm-thick SDC film on LAO reveals an average out-of-plane tensile strain of 0.37%. Comparing this value with the theoretical lattice mismatch of 0.49% between the two materials, we conclude that ∼25% of the theoretical lattice mismatch is lost, most probably through interfacial misfit dislocations. Accordingly, the *in situ* MOSS measurement (Fig. 2a) shows in this case a constant compressive in-plane stress. In accordance to the slightly smaller strain value measured out-of-plane on LAO compared with NGO, the MOSS shows a smaller value (∼3 GPa) of average residual and effective stress for the same total film thickness.

When a strained epitaxial film grows, elastic energy is accumulated until it becomes energetically favourable for dislocations lines to move through the crystal, relieving the strain^9^,^10^,^11^. Experimentally, mismatch values up to around 1% can be expected to be fully accommodated^11^. With the materials and growth condition selected here for SDC, 0.4–0.5% strain was induced starting from a theoretical lattice mismatch of ∼0.5–0.6%. One possible explanation for the stress relaxation could be the growth mechanism. During the SDC growth on all the three different substrates, RHEED did not show a two-dimensional layer-by-layer growth. Instead, RHEED revealed a three-dimensional growth mode, also known as island or layer-plus-island growth mode. Such a growth mechanism along the (001) crystallographic orientation of ceria films has been reported and explained^29^. This growth mode facilitates the nucleation of dislocations^12^,^13^, which assist the strain relaxation.

### Tensile strained SDC films for electrical characterization

To allow a reliable in-plane electrical characterization of thin ion conducting films, the growth platform must add a negligible contribution to the total conductance of the sample. Among the substrates used in this study, only MgO fulfils this condition. As an example, at around 600 °C, the resistance of an NGO substrate is similar to that of a 10-nm-thick SDC film with the same area^30^. However, although MgO is an ideal substrate for conductivity measurements, it only allows the growth of fully relaxed SDC films.

In previous studies, SrTiO_3_ (STO) was used as a thin epitaxial buffer layer on MgO to favour a highly ordered growth of ceria films on an insulating substrate^31^,^32^. More recently, it was found that an additional thin inter-layer of BaZrO_3_ (BZO) with a thickness of 5 nm grown between MgO and STO improves the crystalline quality of the STO layer^33^. The same sample design described in ref. 33 was used here. The 5-nm-thin STO seed layer does not significantly affect the conductance of the sample while providing a lattice mismatch of ∼1.6% (for the fully relaxed structure) with respect to SDC, which will induce an in-plane tensile strain in epitaxially grown SDC.

**Figure 3:**
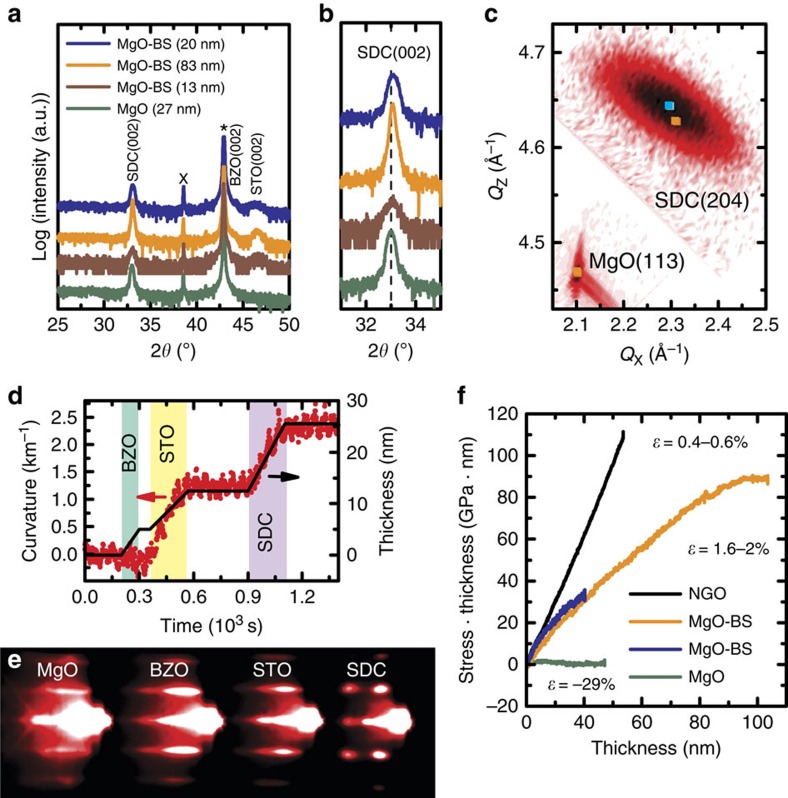
XRD, MOSS and RHEED analysis of SDC on MgO+BZO+STO. (**a**) *ω*/2*θ* scans. * and X indicate the substrate peak and an instrumental artefact (*K*_β_ reflection of substrate), respectively. (**b**) Magnification of the region around the (002) diffraction peak of SDC. The stress-free film on MgO is included for comparison. (**c**) RSM of the (204) reflection of the 20-nm SDC film. The (113) asymmetric diffraction of the MgO substrate was used for alignment. Orange square indicates the unstrained literature values and blue square the centre of the SDC(204) reflection. (**d**) representative MOSS measurement of wafer curvature during the growth of a complete heterostructure and (**e**) simultaneous RHEED measurement. For the RHEED diagnostic, the electron beam is parallel to the (110) direction of the substrate, thus parallel to (110) of BZO and STO, which show a cube-on-cube growth, but parallel to (100)—equivalently (010)—direction of SDC due to the in-plane 45° rotation of the unit cell of the ionic conductor with respect to the template platform. (**f**) MOSS measurements during the growth of the SDC films with different stress states.

Figure 3a shows the XRD analysis of three epitaxial SDC films, 13, 20 and 83 nm-thick, grown on the (001)-oriented MgO+BZO+STO template platform (hereafter we will refer to this template platform as MgO-BS). In spite of the small thickness of the layer, the *ω*/2*θ* scans allow a rough estimation of the out-of-plane compressive strain of the STO layer between 0.1 and 0.5%, thus the misfit between the growth platform and SDC is expected to be in the range of 1.7–2.1% in-plane.

The BZO+STO layers were deposited at around 765 °C. The 20- and 83-nm-thick SDC films on MgO-BS were deposited at 650 °C, the same temperature used for the films on MgO, NGO and LAO. To probe the effect of temperature on the developed stress, the 13-nm-thick film was grown at the same temperature used for BZO and STO.

The shift of the diffraction peak of these three films towards larger 2*θ* angles indicates different out-of-plane compressive strain values (Fig. 3b). To quantify the strain in-plane, reciprocal space maps (RSMs) were recorded. As an example, the RSM acquired along the (204) asymmetric diffraction of the 20-nm-thick SDC film grown on MgO-BS is reported in Fig. 3c. The 20-nm-thick film shows the largest in-plane tensile strain, that is, 0.35%.

As expected, the crystalline structure relaxes with increasing thickness: for the 83-nm film, an in-plane strain of 0.24% is found. The 13-nm-thick film grown at the higher temperature shows the smallest in-plane tensile strain value of ∼0.1%. This can be explained by considering that for a film to relax dislocations have to nucleate or existing dislocation lines have to be mobile. The mobility of a dislocation can be described by a periodic energy barrier that can be overcome by accumulating elastic energy or by providing thermal energy^9^, which facilitates the relaxation process.

Figure 3d, e shows the results of the *in situ* MOSS plus RHEED measurements performed during the growth of the SDC films on the MgO-BS template platform. Figure 3d reports an example of the wafer curvature measurements showing the capability of MOSS diagnostic to probe *in situ* the in-plane stress of the different layers during the growth of a complex heterostructures.

Owing to the very good lattice matching between MgO and BZO, no significant wafer curvature (stress) can be observed during the growth of the 5-nm-thick BZO layer. On the contrary, the lattice mismatch between BZO and STO (as large as 7%) induces an in-plane tensile stress to the STO film, as revealed by the positive wafer curvature measured by MOSS. It is worth highlighting that for such thin layers the MOSS allows the stress state of the film to be unambiguously identified *in situ* much more effectively than by evaluating *ex situ* the angular position of the broad peaks in the *ω*/2*θ* scans. Within this experiment, the MOSS diagnostic allows resolving the change of the substrate curvature induced by the deposition of films with thicknesses in the ⩾1 nm range.

Finally, the substrate continues to bend in the same direction during the growth of the SDC film on top of STO indicating an in-plane tensile strain of the oxygen ion conductor.

The simultaneous RHEED measurements are shown in Fig. 3e. The RHEED patterns of BZO and STO indicate an almost two-dimensional growth. But, as previously discussed, the RHEED pattern for SDC clearly shows a three-dimensional growth mode from the very beginning of the film nucleation.

Figure 3f shows the *in situ* MOSS measurements of the 20- and 83-nm-thick SDC films grown on MgO-BS, as well as those of the SDC films grown on NGO and MgO for comparison. The data for the 20- and 83-nm-film grown with the same deposition parameters on MgO-BS are in good agreement. At the thickness of 20 nm, the MOSS allows estimating an average in-plane stress of ∼1.5 GPa for both films.

The extent of the average tensile stress retained in the SDC layer on MgO-BS was smaller than on NGO (and on LAO in compressive stress). The much larger lattice misfit and the presence of a larger density of crystalline defects in the double buffer layer as compared with a single-crystal substrate can explain the larger stress relaxation.

The stress-thickness curve shows a constant slope up to  65 nm for increasing film thickness until the curve becomes flat between 70 and 75 nm. A constant curvature indicates that the total elastic energy in the film remains constant even though the thickness increases. This can be related to theoretical models for epitaxial thin film relaxation^9^,^10^,^11^ according to which above a critical thickness enough elastic energy is accumulated that it becomes favourable for dislocation lines to move. The average stress is then gradually reduced and the total elastic energy remains constant while the film continues to grow. In other words, MOSS measurements allow identifying *in situ* the onset of stress relaxation in the film.

The measured ratio of stress (MOSS) over strain (RSM), equal to *Y*/(1-*ν*), is the same for both the 20- and 83-nm-thick films and in agreement with the literature^34^ showing that the MOSS indeed reflects directly the average stress of the film.

**Figure 4:**
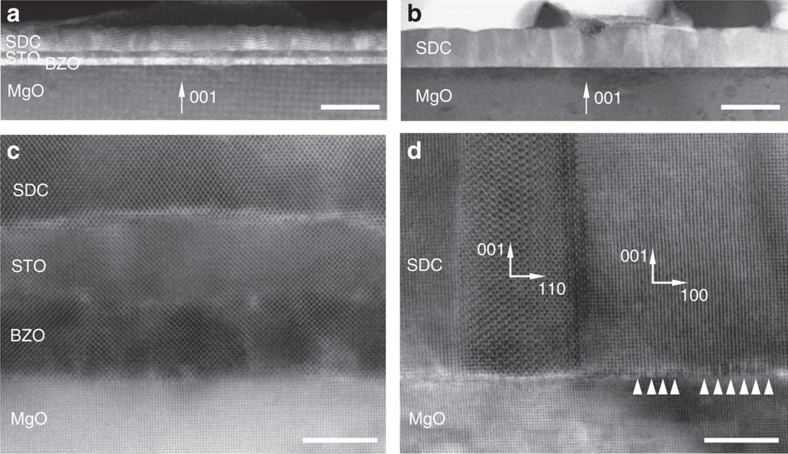
TEM analysis of SDC on MgO-BS (left) and on MgO (right). Low-magnification cross-sectional HAADF-STEM image of (**a**) ∼21-nm-thick SDC film on MgO-BS and (**b**) ∼32-nm-thick SDC film on MgO. Scale bars correspond to 50 nm (**a**), 50 nm (**b**), 5 nm (**c**), 5 nm (**d**). Bright-field high-resolution STEM image of the SDC film on MgO-BS (**c**) and on MgO (**d**). One of the local defects (tilted grains) is shown in **d** together with the positions, marked with arrowheads, of some interfacial misfit dislocation at the SDC/MgO interface. The TEM micrographs were acquired along [010] direction of MgO.

The morphology and microstructure can both influence the evolution of the strain and the activation energy for oxygen ion conduction^35^. The local microstructure of the samples selected for the electrical characterization was investigated using transmission electron microscopy (TEM). Figure 4a, b shows representative examples of SDC films grown on MgO-BS and MgO, respectively.

Figure 4c shows a high-resolution bright-field (BF) STEM image of the cross-section of a SDC film grown on MgO-BS. Selected area electron diffraction and fast Fourier transform analysis reveal very good crystallographic quality showing that the [001] zone axis of the SDC film slightly precesses within only ∼1° on the scales of the order of 100 nm. The SDC film grown directly on the MgO (Fig. 4d) demonstrates a cube-on-cube growth in spite of the very large lattice misfit as already reported in the literature^36^,^37^. Further, the film shows the presence of local isolated domains rotated by 45° around the substrate surface normal. These isolated defects are not present in the film on MgO-BS. The very large lattice mismatch between SDC and MgO results in a lower crystallographic quality of the SDC film, which shows an average grain size of 40 nm and the SDC/MgO interface characterized by an almost continuous line of misfit dislocations that release the stress still preserving the epitaxial relation. The same kind of interface was observed between Gd-doped ceria and MgO (ref. 38). As reported in ref. 33, this difference in morphology does not influence the ionic conduction. These measurements support the XRD and MOSS results that showed a fully relaxed (001) oriented SDC structure for the thin film grown on MgO and the presence of a measurable in-plane tensile strain for the films grown on MgO-BS.

### Influence of tensile strain on ionic conductivity

**Figure 5:**
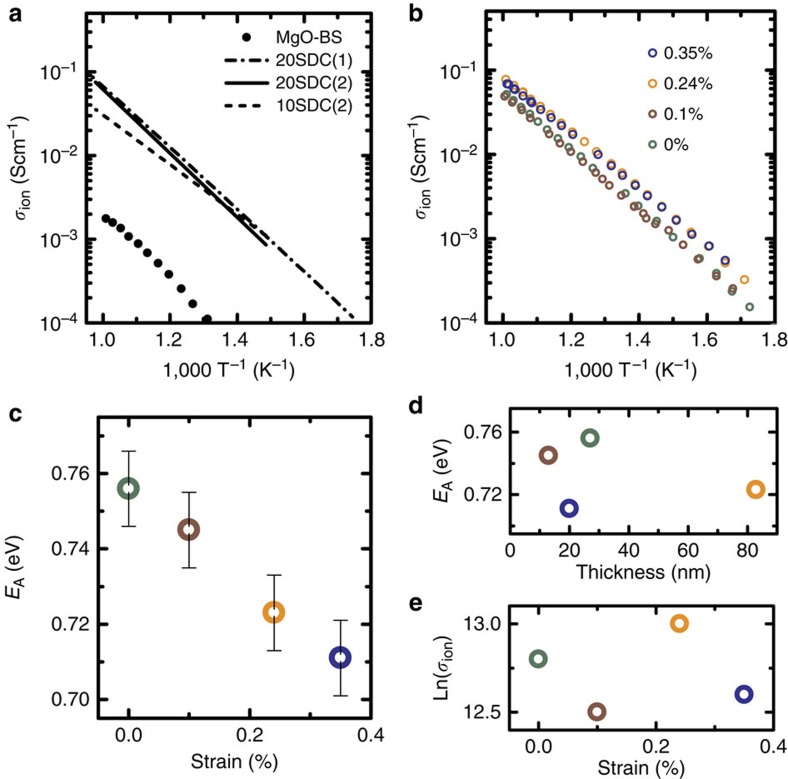
Impedance spectroscopy measurements of SDC films with different strain. (**a**) Conductivity measurement of the MgO+BZO+STO (MgO-BS) template platform compared with literature references of unstrained Sm-doped ceria bulk: 20 at.% Sm (20SDC(1) (ref. 39)) and 10 and 20 at.% Sm (10SDC(2) and 20SDC(2) (ref. 40)). (**b**) Comparison of conductivity measurements of the SDC films fabricated for this study with different in-plane tensile strain values. (**c**) Activation energy for ion migration as a function of tensile strain. The error bar is determined form the standard deviation of the fit and empirically by comparison of independent measurements. (**d**) Activation energy versus SDC thickness and (**e**) Arrhenius pre-exponential factor of the SDC films with different strain values.

AC impedance spectroscopy was applied for electrical characterization using patterned Pt electrodes on the film surface. Figure 5a shows the grain interior (bulk) ionic conductivity of three different Sm-doped ceria samples from the literature^39^,^40^ compared with the measured conductivity of the MgO-BS template platform, which is more than two orders of magnitudes smaller, that is, negligible. Figure 5b shows the electrical characterization of SDC films with different strain values. Activation energy and conductivity values are in very good agreement with reference data for SDC grain interior^39^,^40^,^41^, showing that the grain interior contribution dominates. The complex impedance plane plots (Supplementary Discussion and Supplementary Fig. 1) show a clear polarization of the Pt electrodes at low frequencies, indicating that the dominant charge carriers are indeed oxygen ions, as is well established for SDC under the selected experimental conditions. This leaves no doubt about the nature of the charge carriers.

The activation energy, calculated by fitting the linearized Arrhenius equation (3) to the data, shows a clear trend with respect to the film strain, that is, it decreases with increasing tensile strain (Fig. 5c). Interface effects (for example, dislocations^42^) could also influence the activation energy, resulting in a thickness dependence of the activation energy. This is not the case here (Fig. 5d) suggesting that it is indeed the strain that influences the activation energy. Further, the pre-exponential factor *σ*_*0*_ of the Arrhenius equation varies little (Fig. 5e), showing that the charge carrier density is not affected by the strain.

The change of conductivity is negligible in the high-temperature range, whereas for example at 350 °C (a temperature relevant for miniaturized SOFCs), the bulk ionic conductivity is a factor of two larger compared with the unperturbed crystalline structure as a result of 0.35% tensile lattice strain, which lowers the activation energy by ∼0.05 eV.

The observed influence of the strain values on the activation energy is very similar to that reported in a theoretical study on undoped ceria^43^. From the experimental point of view, literature reports on the effect of strain on conductivity for epitaxial doped ceria films are scarce. In ref. 44, for example, a very large increase in conductivity with increasing tensile strain was reported. Here a surprisingly large value of strain of 2% was estimated for 10 at.% Gd-doped ceria films as thick as 250 nm. However, in this study, larger strain results in higher ionic conductivity, but also in an increased defect density, yielding a much higher activation energy. The same material coupled with Er_2_O_3_ was used for the fabrication of multi-layered microdots (111)-oriented on sapphire^45^. The reported activation energy of the single doped-ceria layer is in good agreement with the typical values reported for this material, even though the total conductivity is surprisingly smaller than the typical bulk conductivity of Gd-doped ceria^39^,^41^ suggesting that conduction pathways other than through the bulk dominate. However, the authors reported that a change of the compressive in-plane strain of the ceria layers from about 0.1 to 1.1% results in an increase of the activation energy by 0.2 eV. Assuming an almost linear trend of activation energy versus strain for strain values within±1%, the reported effect in ref. 45 is in line with our measurements.

Comparing the effect on SDC to a different oxygen ion conductor, that is, yttria-stabilized zirconia (YSZ), a similar increase in conductivity can be extrapolated for this particular strain value (0.35%) from a theoretical study on the influence of strain in YSZ^46^. The result reported here is also in agreement with the ∼3.4-fold enhanced conductivity measured for YSZ/Y_2_O_3_ 0.8% strained multilayers with columnar morphology^47^. Few papers report no effect at all, for example, using CeO_2_/YSZ multi-layered heterostructures^32^, where the typical YSZ bulk conductivity was measured for all samples. In that work, the non-uniform strain along the layers revealed by high-resolution TEM and RSM could not be quantified and the residual and effective strain could be small. Moreover, only the temperature range above 400 °C was investigated, whereas in the present work, impedance measurements were acquired down to a minimum temperature of 280 °C, and it is precisely at lower temperatures where the effect is more evident.

On the contrary, as already pointed out, many other papers report very large strain effect on the ionic conductivity which are not in agreement with this or the above-mentioned studies. Several examples can be found in ref. 8. Finally, the effect of the local strain arising from buckling in free-standing polycrystalline membranes of doped ceria^48^, as eventually relevant for devices, is discussed elsewhere, as well as the effect of strain in polycrystalline samples (sintered pellets or film crystallized by post-annealing), where the origin of strain is the specific local micro-morphology^49^, which is completely different to the almost single crystalline samples in our study.

## Discussion

Our results on the role of strain on the ionic conductivity in Sm-doped ceria shows that, as reported in many literature contributions, lower activation energies indeed result from tensile lattice distortions in the direction of the migration of the charge carriers. Our study was conducted on samples as closely resembling a uniformly strained single crystal as it was experimentally possible showing the characteristic bulk conductivity of the material under investigation.

It should be highlighted that the in-plane geometry of the electrical characterization dictates stringent limitations to the choice of the substrate. As a consequence, a relatively small value of residual and effective strain could be preserved. However, it was shown that significantly larger strain can be achieved. As an example, extrapolating the observed effect of strain on the activation energy, around 350 °C, a 0.5% tensile strained ion conductor, as achieved for films grown on NGO, would show the same conductivity as the unstrained conductor at around 450 °C.

Beside the specific application to oxygen ion conductors, which supports the thesis of a relatively small effect of strain on ionic conductivity, the most important result of our study is that it has been clearly shown that the MOSS is a valuable tool for investigating *in situ* the mechanism of stress generation and evolution in complex multi-layered oxide heterostructures. The stress states arising for different lattice mismatches as well as the relaxation behaviour in the epitaxial oxide films grown by PLD could be clearly monitored and were found to be in very good agreement with standard *ex situ* XRD analysis. Moreover, the stress state of the film can be clearly identified also for very thin layers in the nanometre range, well below the limit of conventional XRD analysis. Finally, the MOSS diagnostic of oxide materials has been coupled for the first time with RHEED during PLD.

Strain-controllable ionic or mixed ionic/electronic conductivity can boost the development of oxide heterostructures as active components for micro-electrochemical devices^45^, such as micro-SOFCs^50^, electrochemical sensors, resistive switching memories^51^, or as active catalytic surfaces. But the potential of the MOSS plus RHEED diagnostics for lattice strain engineering goes far beyond this, providing a tool for tuning material properties for a broad range of applications (multiferroicity, catalysis, nano-device fabrication) in many different disciplines opening unprecedented opportunities in Materials Science.

## Methods

### Thin film deposition and *in situ* techniques

The thin films were fabricated in a PLD system from Twente Solid State Technology with integrated MOSS and RHEED systems (k-Space Associates, Inc.). The vacuum chamber, equipped with load-lock chamber, has a base pressure of about 10^−8^ mbar. Gas inlet lines allow setting the required background partial pressure during the deposition. A radiant resistive heater sets the desired deposition temperature. The substrate temperature was monitored with a pyrometer. The target carousel can hold up to five different targets. A custom-made substrate holder allows the simultaneous alignment of the substrate surface with the ablation plume, the RHEED and the MOSS systems.

10 × 10 × 0.5 mm^3^ substrates from CrysTec GmbH are used. For MgO, a special substrate preparation is required where the substrates are cut in small wafers after polishing in order to obtain negligible surface curvature. This procedure results in a not atomically flat surface, as revealed by RHEED. By annealing the substrates in O_2_ at 1,000 °C for 12 h, two-dimensional surface reconstruction was obtained. The substrate holder supports the substrate only in the corners without mechanical constrain to allow it to bend freely. As the MgO substrates have a transmission wavelength range of 0.2–8 μm, to ensure an accurate temperature reading and an efficient substrate heating, the unpolished surface of the substrates is coated with Pt (sputtered with 40 W at 3 × 10^−2^ mbar for 4 min, ∼400 nm) used as heat absorber. Such a thin layer does not affect the overall elastic properties of the substrate; neither has it influenced the MOSS measurements of the relative changes in the substrate curvature.

Ceramic sintered pellets of BZO, STO and 15 at.% SDC, prepared in our laboratories, were ablated using a 248-nm KrF excimer laser (Coherent Lambda Physics GmbH) with pulse lenght of 25 ns. A spot size of the laser on the target of 1.2 mm^2^, a fluence of 1.2 J cm^−2^, a frequency of 4 Hz and a target to substrate distance of 5 cm were used. The oxygen back ground pressure was set to 5 × 10^−2^ mbar. BZO and STO were deposited at ∼740 °C and SDC, unless otherwise indicated, at 650 °C. The deposition rates have been accurately calibrated by X-ray reflectometry to be 0.14 Å pulse^−1^ for BZO, 0.09 Å pulse^−1^ for STO and 0.11 Å pulse^−1^ for SDC. The samples were well reproducible, for example, concerning crystallinity, stress state, conductive properties and so on.

For the *in situ* wafer curvature measurement with the MOSS, a 3 × 3 array of laser beams is used. Typically, the measured wafer curvature is converted into the stress-thickness product via Stoney's equation and plotted as a function of thickness (see Supplementary Methods for more details; Supplementary Fig. 2). The main advantages of MOSS over similar techniques, for example, using a cantilever as substrate, are that any substrate can be used, no position-sensitive detector is needed, and the noise is reduced through multiple beams.

### *Ex situ* characterization

XRD characterizations were performed with a Siemens D500 diffractometer. The RSMs were recorded for the MgO(113) and the SDC(204) diffraction peak separately with the integration time for the film peak being 20 times higher. The RSMs consist of rocking curves, recorded for a range of 2*θ* values. The centre of the diffraction is determined by fitting individual rocking curves in the software Diffract.EVA.

A difference in the thermal expansion coefficients could lead to different *in* and *ex situ* strain values. However, the linear expansion coefficients for the materials used in this work are all around 10^−5^ K^−1^ (NGO (ref. 52), MgO (ref. 53), LAO (ref. 54), SDC (ref. 55), STO (ref. 54), BZO (ref. 56)) and the expansion mismatch is negligible (for example, 0.003% for SDC grown at 700 °C on MgO).

Cross-sectional TEM specimens were prepared by mechanical polishing and low-voltage Ar^+^ ion milling for the final thinning. TEM and STEM investigations were performed on a Titan 80-300 (FEI) Environmental Transmission Electron Microscope equipped with an imaging-side aberration corrector. The experiments were conducted at an acceleration voltage of 300 kV. Atomic-resolution Z contrast images were obtained by HAADF and BF STEM imaging. The inner and outer collection angles of the detector were 70 and 200 mrad, respectively.

The impedance spectroscopy measurements were carried out under a flow of O_2_ in a tube furnace. Rectangular parallel Pt electrodes were used, defined by stainless steel masks except for the characterization of the growth platform, where interdigitated electrodes were patterned by ultraviolet photolithography (Supplementary Methods and Supplementary Fig. 3). The electrodes were deposited by magnetron sputtering at room temperature. Pt was sputtered with 40 W at 3 × 10^−2^ mbar for 2 min (100–200 nm) on a Ti sticking layer sputtered with 20 W at 7 × 10^−2^ mbar for 1.5 min (∼5 nm). The target to substrate distance was 4 cm. The electrodes were wired to the read-out electronics using Ag paste and Au wires. The impedance was measured using a Solartron 1260 impedance/gain-phase analyser with a bias voltage of 1 V in the frequency range between 1 Hz and 1 MHz, varying the temperature between 300 and 720 °C. Data analysis was performed with EC-Lab (V10.31), where the data were fit to the response of a RC parallel circuit.

## Additional information

**How to cite this article:** Fluri, A. *et al*., *In situ* stress observation in oxide films and how tensile stress influences oxygen ion conduction. 7:10692 doi: 10.1038/ncomms10692 (2016).

## Supplementary Material

Supplementary Information Supplementary Figures 1-3, Supplementary Discussion, Supplementary Methods and Supplementary References

## Figures and Tables

**Figure 1:**
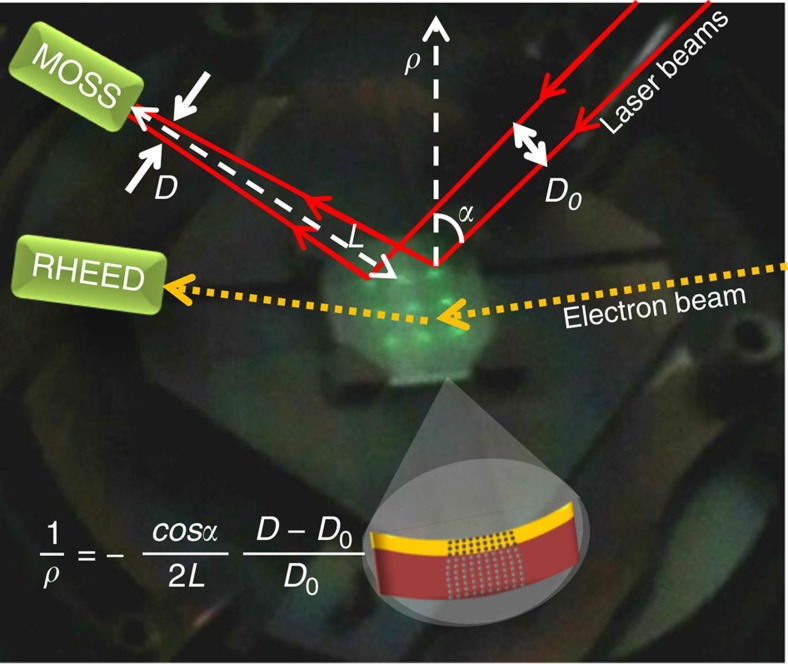
Working principle of the multi-beam optical stress sensor (MOSS). 10 × 10 mm^2^ MgO substrate on the sample holder of the PLD system equipped with MOSS and RHEED. A 3 × 3 array of parallel laser beams (visible as bright spots on the substrate surface) is reflected by the substrate towards a CCD camera that records the relative distance between the laser spots. The paths of two laser beams of the MOSS and of the electron beam of the RHEED are sketched. The growth of a strained layer induces a change of curvature (1/*ρ*) of the substrate and a change of the direction of the reflected laser beams. The effect of a stress-induced curvature of the substrate is illustrated in cross-section for the case of an in-plane tensile strained film (*ρ*>0). The relative curvature change is obtained by measuring the change in the relative distance (*D*−*D*_0_)/*D*_0_ between the laser beams; *D*_0_ being the distance at the beginning of the growth, *L* the optical path length and *α* the incident angle. From the CCD image, the MOSS software calculates the average of (*D*−*D*_0_)/*D*_0_ using all nine beams.

**Table 1:** Characteristics of the single crystal substrates used for this study.

**Substrate**	**Crystalline structure**	**Expected epitaxial relation**		**Lattice mismatch (%)**
NdGaO_3_ (110)	Orthorhombic perovskite *a*=5.43 Å *c*=5.50 Å	In-plane	SDC(1 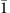 0)||NGO(1 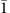 0) SDC(110)||NGO(001)	0.64, 0.39
		Out-of-plane	SDC(001)||NGO(110)	
LaAlO_3_ (001)	Pseudocubic perovskite *a*=3.82 Å	In-plane	SDC(110)||LAO(100) SDC(1 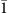 0)||LAO(010)	−0.49, −0.49
		Out-of-plane	SDC(001)||LAO(001)	
MgO (001)	face-centered cubic *a*=4.21 Å		None	≈−29

Expected epitaxial relations and lattice mismatch with 15 at.% Sm-doped CeO_2_ are displayed. The lattice mismatch, given in percent, is calculated as *(a*_substrate_ *– a*_SDC_*)/a*_substrate_.

## References

[b1] Healy N. . Extreme electronic bandgap modification in laser-crystallized silicon optical fibres. Nat. Mater. 13, 1122–1127 (2014). 25262096 10.1038/nmat4098

[b2] Ramesh R. & Spaldin N. A. Multiferroics: progress and prospects in thin films. Nat. Mater. 6, 21–29 (2007). 17199122 10.1038/nmat1805

[b3] Strasser P. . Lattice-strain control of the activity in dealloyed core-shell fuel cell catalysts. Nat. Chem. 2, 454–460 (2010). 20489713 10.1038/nchem.623

[b4] Li S. . Strain-controlled thermal conductivity in ferroic twinned films. Sci. Rep. 4, 6375 (2014). 25224749 10.1038/srep06375 PMC4165272

[b5] Llordés A. . Nanoscale strain-induced pair suppression as a vortex-pinning mechanism in high-temperature superconductors. Nat. Mater. 11, 329–336 (2012). 22327747 10.1038/nmat3247

[b6] Giri G. . Tuning charge transport in solution-sheared organic semiconductors using lattice strain. Nature 480, 504–508 (2011). 22193105 10.1038/nature10683

[b7] Kilner J. A. Ionic conductors: feel the strain. Nat. Mater. 7, 838–839 (2008). 18955995 10.1038/nmat2314

[b8] Yildiz B. “Stretching” the energy landscape of oxides - effects on electrocatalysis and diffusion. MRS Bull. 39, 147–156 (2014).

[b9] Hanbücken M. Stress And Strain In Epitaxy: Theoretical Concepts, Measurements and Applications Elsevier (2001).

[b10] Suresh S. & Freund L. B. Thin Film Materials: Stress, Defect Formation And Surface Evolution Cambridge Univ. (2006).

[b11] Ayers J. E. Heteroepitaxy Of Semiconductors: Theory, Growth, and Characterization CRC Press Taylor & Francis Group (2007).

[b12] Jesson D. E., Pennycook S. J., Baribeau J.-M. & Houghton D. C. Surface stress, morphological development, and dislocation nucleation during strained-layer epitaxy. MRS Online Proc. Library 317, 31–37 (1993).

[b13] Yang W. H. & Srolovitz D. J. Cracklike surface instabilities in stressed solids. Phys. Rev. Lett. 71, 1593–1596 (1993). 10054447 10.1103/PhysRevLett.71.1593

[b14] Zhu J., Li Y. R., Zhang Y., Liu X. Z. & Tao B. W. Effects of compressive and tensile stress on the growth mode of epitaxial oxide films. Ceram. Int. 34, 967–970 (2008).

[b15] Bauer S. . The power of in situ pulsed laser deposition synchrotron characterization for the detection of domain formation during growth of Ba_0.5_Sr_0.5_TiO_3_ on MgO. J. Synchrotron Radiat. 21, 386–394 (2014). 24562560 10.1107/S1600577513034358

[b16] Brown A. S. & Losurdo M. in Handbook of Crystal Growth 2nd Edn ed. Thomas F. Kuech 1169–1209Elsevier (2015).

[b17] Premper J., Sander D. & Kirschner J. *In situ* stress measurements during pulsed laser deposition of BaTiO_3_ and SrTiO_3_ atomic layers on Pt(0 0 1). Appl. Surf. Sci. 335, 44–49 (2015).

[b18] Jacobs R. N. . Dynamic curvature and stress studies for MBE CdTe on Si and GaAs substrates. J. Electron. Mater. 44, 3076–3081 (2015).

[b19] Chason E. . Growth of patterned island arrays to identify origins of thin film stress. J. Appl. Phys. 115, 123519 (2014).

[b20] Scharf T., Faupel J., Sturm K. & Krebs H.-U. Intrinsic stress evolution in laser deposited thin films. J. Appl. Phys. 94, 4273–4278 (2003).

[b21] Floro J. A., Chason E., Cammarata R. C. & Srolovitz D. J. Physical origins of intrinsic stresses in Volmer-Weber thin films. MRS Bull. 27, 19–25 (2002).

[b22] Abadias G., Fillon A., Colin J. J., Michel A. & Jaouen C. Real-time stress evolution during early growth stages of sputter-deposited metal films: influence of adatom mobility. Vacuum 100, 36–40 (2014).

[b23] Chason E., Shin J. W., Hearne S. J. & Freund L. B. Kinetic model for dependence of thin film stress on growth rate, temperature, and microstructure. J. Appl. Phys. 111, 083520 (2012).

[b24] Floro J. A. & Chason E. Measuring Ge segregation by real-time stress monitoring during Si_1−x_Ge_x_ molecular beam epitaxy. Appl. Phys. Lett. 69, 3830–3832 (1996).

[b25] Michotte S. & Proost J. *In situ* measurement of the internal stress evolution during sputter deposition of ZnO:Al. Sol. Energy Mater. Sol. Cells 98, 253–259 (2012).

[b26] Wachsman E. D. & Lee K. T. Lowering the temperature of solid oxide fuel cells. Science 334, 935–939 (2011). 22096189 10.1126/science.1204090

[b27] Jiang J. & Hertz J. L. On the variability of reported ionic conductivity in nanoscale YSZ thin films. J. Electroceram. 32, 37–46 (2014).

[b28] Korte C. . Coherency strain and its effect on ionic conductivity and diffusion in solid electrolytes - an improved model for nanocrystalline thin films and a review of experimental data. Phys. Chem. Chem. Phys. 16, 24575–24591 (2014). 25309994 10.1039/c4cp03055a

[b29] Pergolesi D., Fronzi M., Fabbri E., Tebano A. & Traversa E. Growth mechanisms of ceria- and zirconia-based epitaxial thin films and hetero-structures grown by pulsed laser deposition. Mater. Renew. Sustain. Energy 2, 1–9 (2012).

[b30] Petric A. & Huang P. Oxygen conductivity of Nd(SrCa)Ga(Mg)O_3−δ_ perovskites. Solid State Ionics 92, 113–117 (1996).

[b31] Sanna S. . Fabrication and electrochemical properties of epitaxial samarium-doped ceria films on SrTiO_3_-buffered MgO substrates. Adv. Funct. Mater. 19, 1713–1719 (2009).

[b32] Pergolesi D. . Tensile lattice distortion does not affect oxygen transport in Yttria-stabilized zirconia-CeO_2_ heterointerfaces. ACS Nano 6, 10524–10534 (2012). 23106091 10.1021/nn302812m

[b33] Pergolesi D. . Probing the bulk ionic conductivity by thin film hetero-epitaxial engineering. Sci. Tech. Adv.Mater 16, 015001 (2015). 10.1088/1468-6996/16/1/015001 PMC5036489 27877751

[b34] Wachtel E. & Lubomirsky I. The elastic modulus of pure and doped ceria. Scripta Mater. 65, 112–117 (2011).

[b35] Göbel M. C., Gregori G., Guo X. & Maier J. Boundary effects on the electrical conductivity of pure and doped cerium oxide thin films. Phys. Chem. Chem. Phys. 12, 14351–14361 (2010). 20890498 10.1039/c0cp00385a

[b36] Pérez Casero R. . Epitaxial growth of CeO_2_ on MgO by pulsed laser deposition. Appl.Surf. Sci. 109-110, 341–344 (1997).

[b37] Chen L. . Electrical properties of a highly oriented, textured thin film of the ionic conductor Gd:CeO_2−δ_ on (001) MgO. Appl. Phys. Lett. 83, 4737–4739 (2003).

[b38] Sanna S. . Enhancement of the chemical stability in confined δ-Bi_2_O_3_. Nat. Mater. 14, 500–504 (2015). 25849531 10.1038/nmat4266

[b39] Sameshima S. . Electrical conductivity and diffusion of oxygen ions in rare-earth-doped ceria. Nippon Seramikkusu Kyokai Gakujutsu Ronbunshi/J. Ceramic Soc. Jpn 108, 1060–1066 (2000).

[b40] Giannici F. . Structure and oxide ion conductivity: local order, defect interactions and grain boundary effects in acceptor-doped ceria. Chem. Mater. 26, 5994–6006 (2014).

[b41] Balazs G. B. & Glass R. S ac impedance studies of rare earth oxide doped ceria. Solid State Ionics 76, 155–162 (1995).

[b42] Sun L., Marrocchelli D. & Yildiz B. Edge dislocation slows down oxide ion diffusion in doped CeO_2_ by segregation of charged defects. Nat. Commun. 6, 1–10 (2015). 10.1038/ncomms7294 25723877

[b43] De Souza R. A., Ramadan A. & Horner S. Modifying the barriers for oxygen-vacancy migration in fluorite-structured CeO_2_ electrolytes through strain: a computer simulation study. Energy Environ. Sci. 5, 5445–5453 (2012).

[b44] Mohan Kant K., Esposito V. & Pryds N. Strain induced ionic conductivity enhancement in epitaxial Ce_0.9_Gd_0.1_O_2−δ_ thin films. Appl. Phys. Lett. 100, 033105 (2012).

[b45] Schweiger S., Kubicek M., Messerschmitt F., Murer C. & Rupp J. L. M. A microdot multilayer oxide device: Let us tune the strain-ionic transport interaction. ACS Nano 8, 5032–5048 (2014). 24720562 10.1021/nn501128y

[b46] Kushima A. & Yildiz B. Oxygen ion diffusivity in strained yttria stabilized zirconia: Where is the fastest strain? J. Mater. Chem. 20, 4809–4819 (2010).

[b47] Aydin H., Korte C., Rohnke M. & Janek J. Oxygen tracer diffusion along interfaces of strained Y_2_O_3_/YSZ multilayers. PCCP 15, 1944–1955 (2013). 23258566 10.1039/c2cp43231e

[b48] Shi Y., Bork A. H., Schweiger S. & Rupp J. L. M. The effect of mechanical twisting on oxygen ionic transport in solid-state energy conversion membranes. Nat. Mater. 14, 721–727 (2015). 26076303 10.1038/nmat4278

[b49] Rupp J. L. M. . Scalable oxygen-ion transport kinetics in metal-oxide films: impact of thermally induced lattice compaction in acceptor doped ceria films. Adv. Funct. Mater. 24, 1562–1574 (2014).

[b50] Evans A., Bieberle-Hütter A., Rupp J. L. M. & Gauckler L. J. Review on microfabricated micro-solid oxide fuel cell membranes. J. Power Sources 194, 119–129 (2009).

[b51] Messerschmitt F., Kubicek M., Schweiger S. & Rupp J. L. M. Memristor kinetics and diffusion characteristics for mixed anionic-electronic SrTiO_3-δ_ bits: The memristor-based cottrell analysis connecting material to device performance. Adv. Funct. Mater. 24, 7448–7460 (2014).

[b52] Sasaura M., Miyazawa S. & Mukaida M. Thermal expansion coefficients of high-T_c_ superconductor substrate NdGaO_3_ single crystal. J. Appl. Phys. 68, 3643–3644 (1990).

[b53] Tsay Y.-f., Bendow B. & Mitra S. S. Theory of the temperature derivative of the refractive index in transparent crystals. Phys. Rev. B 8, 2688–2696 (1973).

[b54] Char K., Antognazza L. & Geballe T. H. Study of interface resistances in epitaxial YBa_2_Cu_3_O_7−x_/barrier/YBa_2_Cu_3_O_7−x_ junctions. Appl. Phys. Lett. 63, 2420–2422 (1993).

[b55] Hyodo J., Ida S., Kilner J. A. & Ishihara T. Electronic and oxide ion conductivity in Pr_2_Ni_0.71_Cu_0.24_Ga_0.05_O_4_/Ce_0.8_Sm_0.2_O_2_ laminated film. Solid State Ionics 230, 16–20 (2013).

[b56] Zhao Y. & Weidner D. J. Thermal expansion of SrZrO_3_ and BaZrO_3_ perovskites. Phys. Chem. Minerals 18, 294–301 (1991).

[b57] Guennou M., Bouvier P., Garbarino G. & Kreisel J. Structural investigation of LaAlO_3_ up to 63 GPa. J. Phys. Condens. Matter. 23, 395401 (2011). 21900739 10.1088/0953-8984/23/39/395401

[b58] Krivchikov A. I. . Structure, sound velocity, and thermal conductivity of the perovskite NdGaO_3_. Low Temp. Phys. 26, 370–374 (2000).

[b59] Ahrens T. J. Mineral Physics & Crystallography: A Handbook of Physical Constants American Geophysical Union (1995).

