# Supplementary material for: In situ stress observation in oxide films and how tensile stress influences oxygen ion conduction
Source: Nat Commun. 2016 Feb 25;7:10692. doi: 10.1038/ncomms10692 (PMC4773421; doi:10.1038/ncomms10692)
Supplement: Supplementary Information — Supplementary Figures 1-3, Supplementary Discussion, Supplementary Methods and Supplementary References [file ncomms10692-s1.pdf]

Supplementary information

Supplementary Figures

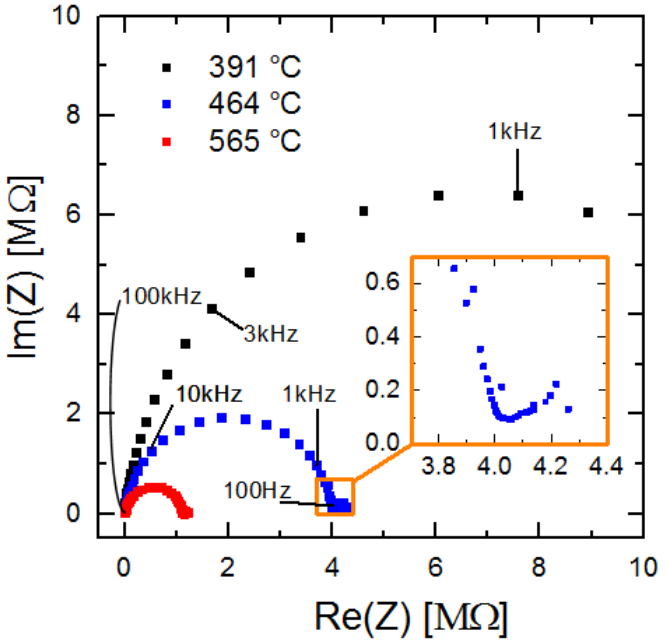

Supplementary Figure 1. Complex Impedance plane plots of SDC film on MgO-BS template platform: Three different temperatures are displayed, with a magnification of the low-frequency part of the 464°C measurement. Selected points are labelled with the respective frequency.

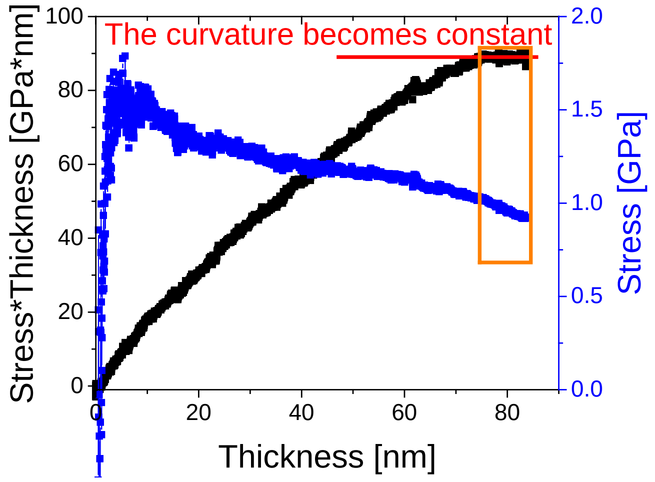

Supplementary Figure 2. Stress*thickness product (black) and average film stress (blue) as a function of thickness.

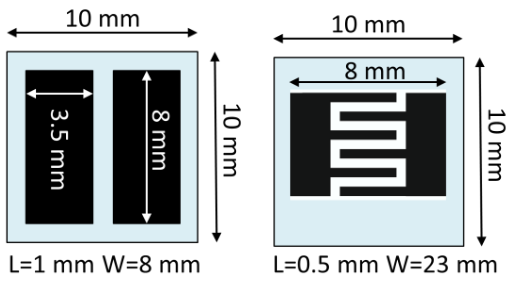

Supplementary Figure 3. Electrode Schematics. Rectangular parallel electrodes (left) and interdigitated electrodes (right): The length L and width W of the conduction channel are indicated. The 10x10 mm^2 square depicts the substrate.

Supplementary Discussion

Impedance Spectroscopy

Supplementary Fig. 1 shows the complex impedance plane plots acquired for an SDC film on the MgO+$BaZrO_3$+$SrTiO_3$ (MgO-BS) growth platform. The polarisation of the electrode is visible, shown in the magnification, at low frequencies. This suggests a blocking effect of the Pt electrodes for the charge carriers providing strong indication that the dominant charge carriers are not electrons or holes, but ions.

With the conductivity and activation energy of the samples being in good agreement with literature data (discussed in manuscript for Fig. 5), the dominant charge carriers are O-ions, as expected for this material in the described experimental conditions. The linearized Arrhenius equation

$$Ln(\sigma T) = Ln(\sigma_0) * (-\frac{E_A}{k_B T})$$

is fitted to the data to obtain the activation energy E_A and the pre-exponential factor as $Ln(\sigma_0)$.

Supplementary Methods

Remarks on the Multi-beam Optical Stress Sensor (MOSS)

The MOSS is a stress measurement technique based on the in-situ measurement of the sample curvature during the growth of a thin film. The working principle of the MOSS is described in detail elsewhere[1]. In brief, the MOSS is a laser deflectometer that illuminates the substrate surface with an array of parallel beams that are reflected from the substrate surface toward a CCD camera which records their relative position. As a consequence of the growth of a thin film in compressive or tensile stress, a force is applied parallel to the substrate surface

inducing a curvature in the substrate. The stress-induced curvature introduces a divergence into the laser array, changing the mean differential spacing $(D - D_0)/D_0$ (see Fig. 1 in manuscript) of the reflected beams. The relative change of the mean differential spacing is proportional to the relative change of substrate curvature which is in turn proportional to the stress-thickness product of the growing film through the Stoney equation. To our knowledge MOSS, RHEED and PLD are combined the first time in our laboratory.

The angle of incidence of the laser beams or their orientation with respect to the crystallographic orientation of the substrate does not affect the measurement of the curvature (see Fig. 1 in manuscript), thus using the Stoney equation the stress measurement is always exactly in-plane, i.e. parallel to the substrate surface.

The stress thickness product is used as a unit, as this is commonly done in papers where a MOSS is used[2-8], stress being the average stress over the film thickness. The advantages of this unit are:

- It is directly proportional to the raw measurement data, i.e. the curvature
- As compared to a curvature plot, the stress values can be read out relatively easily. More importantly, while for one particular strain value of the film the resulting curvature depends the elastic constant of the substrate (see Stoney's equation), the stress*thickness product does not.
- As the stress is the average over the whole thickness, immediate changes are better visible in the stress*thickness product, e.g. the point where the curvature, and thus stress*thickness becomes constant (red line in Supplementary Fig. 2). In a stress plot this is only visible as a slight decrease in stress (orange square in Supplementary Fig. 2). After this point, the total elastic energy of the entire film remains constant while the thickness increases, which can be related to models for thin film relaxation[1,9,10].

Electrode Geometry

The electrode schematics are shown in Supplementary Fig. 3 with a distance between the electrodes of 1 or 0.5 mm and a film thickness of tens of nm, the electric field can be safely assumed to be purely in-plane and uniform across the film thickness. Interdigitated electrodes were only used for the electrical characterization of the template platform MgO + $BaZrO_3$ + $SrTiO_3$ due to its high resistance.

Supplementary References

1 Suresh, S. & Freund, L. B. *Thin Film Materials: Stress, Defect Formation And Surface Evolution* (Cambridge University Press, Cambridge, 2006).

2 Chason, E. et al. Growth of patterned island arrays to identify origins of thin film stress. *Journal of Applied Physics* **115** (2014).

3 Scharf, T., Faupel, J., Sturm, K. & Krebs, H.-U. Intrinsic stress evolution in laser deposited thin films. *Journal of Applied Physics* **94**, 4273-4278 (2003).

4 Floro, J. A., Chason, E., Cammarata, R. C. & Srolovitz, D. J. Physical origins of intrinsic stresses in Volmer-Weber thin films. *MRS Bulletin* **27**, 19-25 (2002).

5 Abadias, G., Fillon, A., Colin, J. J., Michel, A. & Jaouen, C. Real-time stress evolution during early growth stages of sputter-deposited metal films: Influence of adatom mobility. *Vacuum* **100**, 36-40 (2014).

6 Chason, E., Shin, J. W., Hearne, S. J. & Freund, L. B. Kinetic model for dependence of thin film stress on growth rate, temperature, and microstructure. *Journal of Applied Physics* **111**, (2012).

7 Floro, J. A. & Chason, E. Measuring Ge segregation by real-time stress monitoring during $Si_{1-x}Ge_x$ molecular beam epitaxy. *Applied Physics Letters* **69**, 3830-3832 (1996).

8 Michotte, S. & Proost, J. In situ measurement of the internal stress evolution during sputter deposition of ZnO:Al. *Solar Energy Materials and Solar Cells* **98**, 253-259 (2012).

9 Hanbücken, M. *Stress And Strain In Epitaxy: Theoretical Concepts, Measurements and Applications* (Elsevier, Amsterdam, 2001).

10 Ayers, J. E. *Heteroepitaxy Of Semiconductors: Theory, Growth, and Characterization* (CRC Press Taylor & Francis Group, Boca Raton, 2007).
